# Cancer-causing *BRCA2* missense mutations disrupt an intracellular protein assembly mechanism to disable genome maintenance

**DOI:** 10.1093/nar/gkab308

**Published:** 2021-05-12

**Authors:** Miyoung Lee, David Shorthouse, Robert Mahen, Benjamin A Hall, Ashok R Venkitaraman

**Affiliations:** Medical Research Council Cancer Unit, University of Cambridge, Hills Road, Cambridge CB2 0XZ, UK; Medical Research Council Cancer Unit, University of Cambridge, Hills Road, Cambridge CB2 0XZ, UK; Medical Research Council Cancer Unit, University of Cambridge, Hills Road, Cambridge CB2 0XZ, UK; Medical Research Council Cancer Unit, University of Cambridge, Hills Road, Cambridge CB2 0XZ, UK; Medical Research Council Cancer Unit, University of Cambridge, Hills Road, Cambridge CB2 0XZ, UK; The Cancer Science Institute of Singapore, National University of Singapore, 14 Medical Drive, Singapore 117599 & Agency for Science, Technology and Research (A*STAR), 8A Biomedical Grove 138648, Singapore

## Abstract

Cancer-causing missense mutations in the 3418 amino acid BRCA2 breast and ovarian cancer suppressor protein frequently affect a short (∼340 residue) segment in its carboxyl-terminal domain (DBD). Here, we identify a shared molecular mechanism underlying their pathogenicity. Pathogenic BRCA2 missense mutations cluster in the DBD’s helical domain (HD) and OB1-fold motifs, which engage the partner protein DSS1. Pathogenic - but not benign – DBD mutations weaken or abolish DSS1-BRCA2 assembly, provoking mutant BRCA2 oligomers that are excluded from the cell nucleus, and disable DNA repair by homologous DNA recombination (HDR). DSS1 inhibits the intracellular oligomerization of wildtype, but not mutant, forms of BRCA2. Remarkably, DSS1 expression corrects defective HDR in cells bearing pathogenic BRCA2 missense mutants with weakened, but not absent, DSS1 binding. Our findings identify a DSS1-mediated intracellular protein assembly mechanism that is disrupted by cancer-causing BRCA2 missense mutations, and suggest an approach for its therapeutic correction.

## Graphical Abstract

**Graphical Abstract:**
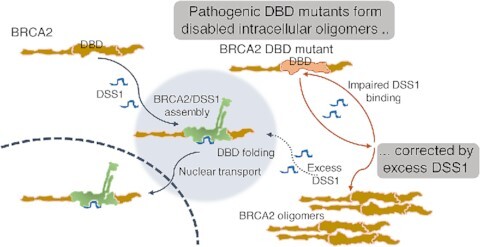
Cancer-causing mutations in the BRCA2 DBD impair DSS1 binding, provoking intracellular oligomerization and cytosolic mis-localization of the mutant BRCA2, and defective homologous recombination. These defects are reversed by DSS1 overexpression, for certain DBD mutants that retain small amounts of DSS1 binding.

## INTRODUCTION

Germline mutations affecting *BRCA2* increase susceptibility to breast, ovarian, prostatic and pancreatic cancers (1–5). Over 12 000 different pathogenic mutations and benign variants have been reported across the entire ∼100 kB span of the human *BRCA2* gene [ClinVar database (https://www.ncbi.nlm.nih.gov/clinvar/)]. Mutations that truncate the encoded BRCA2 protein through frameshift, nonsense codon insertion, or altered splicing are largely considered pathogenic (6,7). However, >50% of *BRCA2* mutations described in the ClinVar database are missense mutations that cause non-synonymous alterations in the BRCA2 protein. Over 90% of these mutations are either classified as variants of unknown significance (VUS), or come with conflicting interpretations of pathogenicity, raising a major clinical challenge for the management of risk in mutation carriers. International collaborative efforts (6–12) combining parameters as wide-ranging as evolutionary conservation of affected residues, or multifactorial likelihood estimates from affected families, have analysed 143 BRCA2 missense mutations via expert panel review to assess pathogenicity with a stringent level of supporting evidence (13). We have investigated here the molecular mechanisms by which a frequently occurring group of these panel-reviewed mutations might affect the biological functions of BRCA2 to cause pathogenicity.

Available evidence indicates that BRCA2 plays an essential role in maintaining human genome integrity through its roles in four biological processes (reviewed in (14)). It controls the localization and assembly on DNA substrates of the recombination enzyme, RAD51, during DNA repair by homologous DNA recombination (HDR) (15–18); protects the structure of stalled DNA replication forks (19,20); suppresses unscheduled RNA-DNA hybrid (R-loop) formation through the regulation of nuclear RNA export and/or promoter-proximal RNA polymerase II pausing (21,22); and has been implicated directly or indirectly in events leading to chromosome segregation (23,24). Moreover, non-homologous end-joining is activated in BRCA2-deficient cells via an Artemis-dependent mechanism (25), but there is little evidence that BRCA2 is a direct mediator of this repair process.

BRCA2 is localized to its major site of activity via nuclear localization signals (NLSs) at its extreme carboxyl-terminus (26,27), but contains few other protein motifs of known function. It may instead work as a hub for the assembly of macromolecular complexes that maintain genome stability through the coordination of different biological processes (reviewed in (14)). For instance, eight conserved sequence motifs of ∼35 residues each (the BRC repeats) in human BRCA2, interact with RAD51, and regulate the assembly of ordered nucleoprotein filaments on DNA substrates (15–17,28–31). The N-terminal region of BRCA2 engages a chromatin-bound partner, PALB2, which recruits BRCA2 and RAD51 to sites of DNA damage (32). Notably, a carboxyl-terminal domain (DSS1/DNA-binding domain; DBD) spanning residues 2457–3184 in BRCA2 binds to a small (∼70 residue) acidic partner, DSS1, to form structures that mediate binding to single- (ss-) or double- (ds) stranded DNA. A crystallographic structure of the BRCA2 DBD reveals that DSS1 binds to the helical domain (HD), and the first (OB1) of three oligonucleotide-/oligosaccharide- binding OB folds. OB2 and OB3 engage ssDNA, while the so-called tower domain contains a helix-loop-helix motif capable of dsDNA-binding (33). DSS1 binding to the BRCA2 DBD has been shown to be essential for its solubility and stability (33–35), suggestive of a role in protein folding. The DSS1/DBD complex is reported to be essential for the nuclear transport of BRCA2/RAD51 complexes (36), and loading of RAD51 on RPA-coated ssDNA to initiate HDR (33,37,38).

Here, we have analysed 9 known pathogenic BRCA2 missense mutations, and report that a common molecular mechanism underlies their pathogenicity. Remarkably, 8 of these 9 pathogenic mutations cluster within the HD and OB1 fold of the BRCA2 DBD, with 1, R3052W, at the OB2/OB3 junction. We find that all these mutations diminish the DSS1/BRCA2 DBD interaction, and present evidence that they disrupt a DSS1-mediated protein assembly mechanism required for nuclear import, to disable HDR. Interestingly, expression of DSS1 can ameliorate defective nuclear transport and correct HDR in cells bearing several hypomorphic BRCA2 missense mutations that diminish but do not ablate DSS1 binding. Collectively, our findings identify a shared molecular mechanism that underlies the pathogenicity of BRCA2 missense mutants that cluster in the DBD and suggest an approach for their therapeutic correction.

## MATERIALS AND METHODS

### Cell culture and transfections

HeLa Kyoto (from the laboratory of Jonathan Pines) and 293T (ATCC) cells were cultured in DMEM (Gibco) supplemented with 10% FBS (Gibco) and maintained at 37°C with 5% CO_2_. JetPRIME transfection reagent (Polyplus Transfection, 114-07) was used for all transfections according to the manufacturer's instruction.

### Molecular cloning and mutagenesis

For mammalian expression, GFP-BRCA2^CT^ plasmids were made by cloning a 3 kb *BRCA2* cDNA fragment encoding residues 2432–3418 into the XhoI/SalI sites of the pEGFP C1 vector (Clontech). DBD mutations were introduced by PCR using two complementary primers (∼40nt) containing mutated sequences and DpnI digestion of the template before transformation of DH5α. The 0.21 kb human DSS1 cDNA was cloned into *Xba*I site of pcDNA3.1 Hygro 5x Myc vector to make the Myc-DSS1 plasmid. mCherry- and paGFP- BRCA2^CT^ plasmids were made by replacing the GFP sequence of GFP-BRCA2^CT^ plasmids with 0.74 kb mCherry or paGFP cDNA fragment at NheI/BspE1 sites.

For bacterial expression, GST-BRCA2^CT^ plasmids contained 3 kb *BRCA2* cDNA fragments encoding human residues 2432–3418, cloned into the SalI/NotI sites of pGEX-4T3 vector (GE Healthcare). Similarly, GFP-BRCA2^CT^ plasmids were cloned from 3.7 kb cDNA fragments encoding an N-terminal GFP tag fused to human BRCA2 residues 2432–3418 region, which were PCR-amplified from the GFP-BRCA2^CT^ construct (in the pEGFP C1 vector) and cloned into the NdeI/NotI sites of the pET21a vector (C-terminal His-tag, Novagen).

### Generation of mutant cell lines by genome editing

BRCA2 DBD missense mutations were introduced into HeLa Kyoto cells using CRISPR protocols developed by the Zhang lab (39). Briefly, sgRNA sequences targeting W2626 and D2723 residues were designed using GPP sgRNA Designer (https://portals.broadinstitute.org/gpp/public/analysis-tools/sgrna-design). sgRNA sequences were cloned into PX458 vector (Addgene Plasmid #48138). Single-stranded DNA oligonucleotides (ssODNs) containing the mutations and diagnostic restriction enzyme sites were synthesized by Integrated DNA Technologies (IDT) and used as HDR templates (Supplementary Table S3). HeLa Kyoto cells plated in 12-well plates were transfected with 0.5 μg PX458 plasmids expressing sgRNAs/Cas9/GFP and 1 μl of 10 μM HDR templates. Two days after transfection, GFP-positive cells were flow-sorted to enrich transfected cells and single cells were plated in 96-well plates. Clones were screened by PCR amplifying target regions and diagnostic restriction enzyme digestion of the PCR products. Positive clones were confirmed by sequencing of the PCR products and further validated by cloning PCR products into pCR Blunt vector (Invitrogen) and sequencing. Two independent clones per each mutation were validated and used for functional analysis.

To generate a *BRCA2* knock-out (KO) cell line, HeLa Kyoto cells were co-transfected with BRCA2 CRISPR/Cas9 KO Plasmid (h) (sc-400700, Santa Cruz Biotechnology) and BRCA2 HDR Plasmid (h) (sc-400700-HDR, Santa Cruz Biotechnology, selection marker: puromycin resistance, RFP). Single clones resistant to puromycin (1 μg/ml) were isolated and screened by western blotting for BRCA2. sc-400700 is a pool of the three sgRNA plasmids: sc-400700 A (CTGTCTACCTGACCAATCGA); sc-400700 B (ATGTAGCACGCATTCACATA); sc-400700 C (CGATTACCTGTGTACCCTTT).

### Immunoprecipitation and western blotting

For immunoprecipitation, 293T cells transfected with the expression constructs were lysed in the NP40 lysis buffer (50 mM HEPES (pH 7.4), 150 mM NaCl, 0.5% NP-40, 1 mM EDTA, 20 mM β-glycerophosphate, 1 mM DTT and protease inhibitor cocktail (Roche, 11873580001)). 1mg of protein extracts were incubated with 1 μl of α-GFP antibody (rabbit, Clontech, 632592) at 4°C overnight. Immune complexes were collected with Protein A Sepharose 4B beads (Sigma, P9424) and washed 3 times with the NP40 lysis buffer before electrophoresis. For western blotting, samples were loaded on either 3–8% Tris-acetate gels or 4–12% Bis–Tris gels (Invitrogen) and transferred to PVDF membrane (Immobilon-P, Millipore). For detection of Myc-DSS1, membranes were treated with 0.2% glutaraldehyde (Sigma) for 45 min before blocking. Primary antibodies used were α-BRCA2 (Ab-1, clone 2B, Merck Millipore OP95, 1:500; clone 5.23, Merck Millipore, 05-666, 1:2000), α-RAD51 (clone 14B4, GeneTex, GTX70230, 1:1000), α-GFP (clone JL-8, Clontech, 632381,1:1000), α-mCherry (α-dsRed, Clontech, 632496, 1:1000), α-Myc (clone 9E10, Santa Cruz, sc-40, 1:500), and α-MEK2 (BD Transduction, 610235, 1:1000). Membranes were incubated with primary and secondary antibodies (HRP-conjugated) diluted in the blocking solution (5% skimmed milk in TBS/Tween buffer (0.1%Tween20/TBS (50 mM Tris–HCl, 150 mM NaCl))) and developed using ECL reagent (GE Healthcare).

### Nucleocytoplasmic localization (microscopy)

Cells grown on coverslips in six-well plates were transfected with GFP-BRCA2^CT^ plasmids. Twenty-four hours later, cells were fixed with 4% PFA (Agar Scientific) for 10 min and permeabilized with TBS–Triton buffer (0.1% Triton-X100/TBS) for 5 min before mounting with Dako Fluorescence Mounting Medium (Dako S3023) containing DAPI (1.5 μg/ml, Sigma). Images acquired with LSM 880 confocal microscope (Zeiss) were analysed using Cell Profiler Human cytoplasm-nucleus translocation assay pipeline. GFP mean intensity in nucleus was divided by mean intensity in cytoplasm to give nucleocytoplasmic ratio.

### Native gel electrophoresis

Samples were prepared in the native lysis buffer (50 mM HEPES (pH 7.4), 50 mM NaCl, 2 mM MgCl_2_, 0.5% NP40, Benzonase 1 U/μl, proteinase inhibitor cocktail). NativePAGE 5% G-250 Sample Additive (ThermoFisher, BN2004) was added to the samples at a final concentration of 0.125%. Samples were separated on NativePAGE Novex 3–12% Bis–Tris Protein Gels (ThermoFisher, BN1001BOX) for 2 h at 150 V. Dark blue cathode buffer was replaced with light blue cathode buffer during the run. Proteins transferred to PVDF membrane were fixed with 8% acetic acid for 15 min and processed for western blotting.

### Nucleocytoplasmic fractionation

Nuclear and cytoplasmic fractions were prepared from BRCA2 mutant cell lines using NE-PER Nuclear and Cytoplasmic Extraction Reagents (Thermo Fisher Scientific, 78833) according to the manufacturer's instruction. Briefly, pellets of 1 × 10^6^ cells were resuspended in 100 μl of CER I reagent, vortexed, and incubated on ice for 10 min. After addition of 5.5 μl of CER II reagent, samples were vortexed and incubated on ice for 1 min. Samples were centrifuged for 5 min at 16 000 × g to obtain cytoplasmic fractions (supernatant). Pellets (nuclei) were resuspended in 50 μl of NER reagent and incubated on ice for 40 min with vortexing every 10 min. After centrifugation at 16 000 × g for 10 min, nuclear fractions (supernatant) were collected. Nuclear and cytoplasmic fractions were analysed by western blotting.

### mClover Lamin A HDR assay

Cells grown on coverslips in six-well plates were transfected with sgRNA plasmid targeting Lamin A (pUC CBA-SpCas9.EF1a-BFP.sgLMNA, Addgene Plasmid #98971) and donor plasmid (pCAGGS Donor mClover-LMNA, Addgene Plasmid #98970). Three days later, cells were fixed with 4% PFA for 10 min and permeabilized with TBS-Triton buffer for 5 min before mounting with DAPI containing medium. Images acquired with LSM 880 microscope were analysed with ImageJ. HDR positive cells were defined as cells with mean mClover nuclear intensity over a threshold set for each experiment (40,41).

### paGFP nuclear import assay

Cells plated in μ-slide 8 well glass bottom chamber slides (ibidi, 80827) were transfected with paGFP-BRCA2^CT^ plasmids. Before live imaging, media was changed to Leibovitz's L-15 medium, no phenol red (ThermoFischer, 21083027) containing 10% FBS. Photoactivation and imaging were performed with LSM 880 confocal microscope. For photoactivation, bleaching tool in Zen software was used with 405 nm laser at 100% intensity, with 50 iterations. Approximately 4.41 μm^2^ area in cytoplasm was activated. Fluorescence intensity in the activated area and the same size area in nucleus were measured for 3 min in time-lapse imaging. For analysis, after background subtraction, fluorescence intensity was normalized to maximum intensity (intensity at peak) in each cell to obtain relative intensity. Mean relative intensity from 8 to 10 cells per sample was plotted with Graphpad Prism.

### DSS1 depletion

HeLa cells were transfected with DSS1 siRNA (Horizon, Dharmacon siGENOME Human SEM1 siRNA-SMARTpool, M-021353-00-0005) or control siRNA (Horizon, Dharmacon On-TARGET plus Non targeting–pool, D-001810-10-05) using JetPRIME transfection reagent (final siRNA concentration: 30 nM). For nucleocytoplasmic distribution of the GFP-BRCA2^CT^ fragment, HeLa cells were transfected with the siRNAs 24 h before transfecting with the GFP-BRCA2^CT^ construct. For the subcellular fractionation experiment, HeLa cells were transfected with the siRNAs 48 h before fractionation. DSS1 siRNA sequences: siRNA-D-021353-01 (GCACAUGUCUGGGAGGAUA); siRNA-D-021353-02 (GUUAUAAGAUGGAGACUUC); siRNA-D-021353-03 (AGACUGGGCUGGCUUAGAU); siRNA-D-021353-04 (CAAUGUAGAGGAUGACUUC)

### RT-qPCR assay

1 μg of RNA samples extracted from the cell pellets using RNeasy Plus mini kit (Qiagen, 74134) were converted to cDNAs using FastGene Scriptase II cDNA Kit (Geneflow, P8-0054) according the manufacturer's instruction. cDNAs were analyzed by qPCR using LightCycler 480 SYBR Green I Master reagent (Roche, 04887352001). DSS1 mRNA level was normalized to HPRT mRNA level.

Primers for DSS1 cDNA: Forward Primer (GAAAAAGCAGCCGGTAGACTT); Reverse Primer (ATCCCAATTATCCTCCCAGACA)

Primers for HPRT cDNA: Forward Primer (TGACACTGGCAAAACAATGCA); Reverse Primer (GGTCCTTTTCACCAGCAAGCT)

### Purification of BRCA2^CT^ protein fragments

To purify GST-BRCA2^CT^ fragments, *Escherichia coli* BL21(DE3) cells transformed with the GST-BRCA2^CT^ constructs (pGEX-4T3 vector) were induced with 0.1 mM IPTG at 21°C overnight. Bacterial pellets were resuspended in PBS (containing 5 mM DTT + protease inhibitor cocktail) and sonicated on ice for lysis. NP-40 was added to the lysates (final concentration of 0.5%) and the lysates were rotated at 4°C for 30 min. After centrifugation (12 000 × g, for 10 min), the supernatants were incubated with the Glutathione Sepharose 4B beads (GE Healthcare, 17-0756-01) at 4°C overnight. The beads were washed with PBS containing 0.5% NP-40 four times and resuspended in PBS containing 5 mM DTT.

To purify GFP-BRCA2^CT^ fragments, *E. coli* BL21(DE3) cells transformed with the GFP-BRCA2^CT^ constructs (pET21a vector, C-terminal His-tag) were induced with 0.1 mM IPTG at 27°C for 3 h. Bacterial pellets were resuspended in the His-purification buffer (20% glycerol, 50 mM sodium phosphate (pH 8.0), 300 mM NaCl, EDTA-free protease inhibitor cocktail) + 5 mM imidazole) and sonicated on ice for lysis. Triton X-100 was added to the lysates (final concentration of 0.5%) and the lysates were rotated at 4°C for 30 min. After centrifugation (12 000 × g, for 10 min), the supernatants were incubated with the Ni-NTA Agarose beads (Qiagen, 30210) at 4°C overnight. The beads were washed with the His-purification buffer containing 0.5% Triton X-100 twice and then with the His-purification buffer containing 20 mM Imidazole twice. GFP-BRCA2^CT^ fragments were eluted with the His-purification buffer containing 50 mM Imidazole and 250 mM imidazole (three fractions each).

Purified proteins were analysed by Coomassie blue staining and western blotting for GFP (GFP-BRCA2^CT^ fragments) or BRCA2 C-terminal residues (anti-BRCA2 antibody, clone 5.23, Merck Millipore, 05-666, both GST- and GFP-BRCA2^CT^ fragments).

### GST-pull down assay

GST-BRCA2^CT^ fragments bound to the Glutathione Sepharose 4B beads were pre-incubated in the binding buffer (150 mM NaCl, 0.5% NP-40, 50 mM Tris.Cl (pH 7.4), 1 mM EDTA, 0.1% BSA, protease inhibitor cocktail) at room temperature for 20 min. After adding the GFP-BRCA2^CT^ fragments, the beads were incubated at room temperature for 1 h before washing with the binding buffer four times. Bound proteins were analysed by western blotting with the anti GFP antibody.

### ΔΔG/RSA calculations

For calculating the ΔΔG/RSA for each mutation/residue, we first curated each crystal structure. Structures were downloaded from the protein data bank, non-protein molecules were removed from the structures, and the structure was relaxed using the Foldx RelaxPDB command within Foldx5 (42). The full command used for relaxation was:

$foldx -command=RepairPDB -pdb=1MJE.pdb -ionStrength=0.05 -pH=7 -vdwdDesign=2

ΔΔ*G* was calculated using Foldx Positionscan command within Foldx5. Positionscan was run on each residue in the structure and the results for residues of interest extracted. The command used for Positionscan was:

$foldx -command=PositionScan -pdb=1MJE repair.pdb -ionStrength=0.05 -pH=7 -vdwdDesign=2 -pdbHydrogens=false -positions=100

for calculating the ΔΔ*G* of each mutation at residue 100.

RSA was calculated using the BioPython python module (43).

### Statistical analysis

Statistical analysis was conducted using Graphpad Prism software (v5). Statistical tests used are listed in the relevant figure legends. *P*-value summary for all statistical tests used in this study is: ns, *P*-value > 0.05; *P-value ≤ 0.05; **P-value ≤ 0.01; ***P-value ≤ 0.001.

## RESULTS

### Clustering of pathogenic BRCA2 missense mutations within the DBD

**Figure 1.**
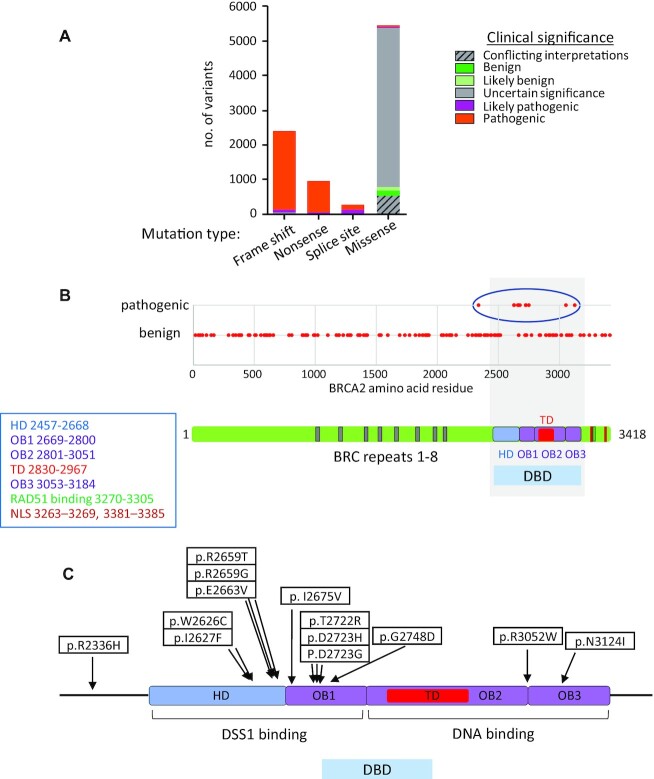
Clustering of pathogenic *BRCA2* missense mutations within the DBD. (**A**) Clinical significance of *BRCA2* mutations by mutation type. The graph shows 9085 variants classified by molecular consequence (mutation type) and clinical significance, out of a total of 12 684 *BRCA2* variants listed in the ClinVar database (version of 2020.07.27). (**B**) Clinical significance of 143 *BRCA2* missense mutations reviewed by the expert panel are shown. Pathogenic mutations clustered in the DBD are marked with a blue circle. The schematic shows human BRCA2 protein structure. Amino acid residue numbers are marked. HD, helical domain: OB, oligonucleotide/oligosaccharide binding; TD, tower domain; NLS, nuclear localisation signal. (**C**) Location of each pathogenic mutation within the BRCA2 DBD.

We analysed 9085 variants of BRCA2 found in the ClinVar database, for which both molecular consequence and clinical significance have been reported (Figure 1A and Supplementary Table S1). Over 50% (5442 variants) encoded missense variants, of which 148 variants were classified as benign, and 37 variants, pathogenic. Assertions of pathogenicity in ClinVar are based on varying levels of evidence (www.ncbi.nlm.nih.gov/clinvar/docs/review_status/). To ensure a stringent threshold of evidence supporting their classification, we confined our analysis to 143 *BRCA2* missense mutations (Figure 1B and Supplementary Table S2) that had been reviewed by an expert panel (https://enigmaconsortium.org/; (13)), excluding mutations affecting the first Met residue in *BRCA2* (which perturb protein translation, c.3G>A (M1I, M231I)). Thirteen of these 143 *BRCA2* mutations were deemed pathogenic by expert panel review (as of 2020.07.27).

Remarkably, 12 of these 13 pathogenic missense mutations cluster within the DBD. By contrast, the remaining missense mutations are distributed across whole length of the BRCA2 protein (Figure 1B and Supplementary Table S2). Moreover, 10/12 pathogenic mutations in the DBD affect the HD and OB1 fold, implicated in DSS1 binding (33), whilst a singleton mutation (R3052W) affects the OB2/OB3 junction, and another singleton (N3124I) falls in OB3 (Figure 1C). We report below mechanistic analyses of 9 pathogenic mutants (W2626C, I2627F, R2659T, E2663V, T2722R, D2723H, D2723G, G2748D and R3052W), which are collectively representative of pathogenic *BRCA2* DBD mutations, and compare them to 2 benign variants (T2515I, K2729N), as well as wild-type BRCA2. Of note, the pathogenic mutant R2659T has been reported to alter *BRCA2* splicing, leading to the in-frame deletion of 57 amino acids encoded in exon 17, and the absence of mutant protein (44); it may not therefore represent a biologically significant *BRCA2* mis-sense mutant.

### Pathogenic BRCA2 mutations in the DBD affect DSS1 binding

**Figure 2.**
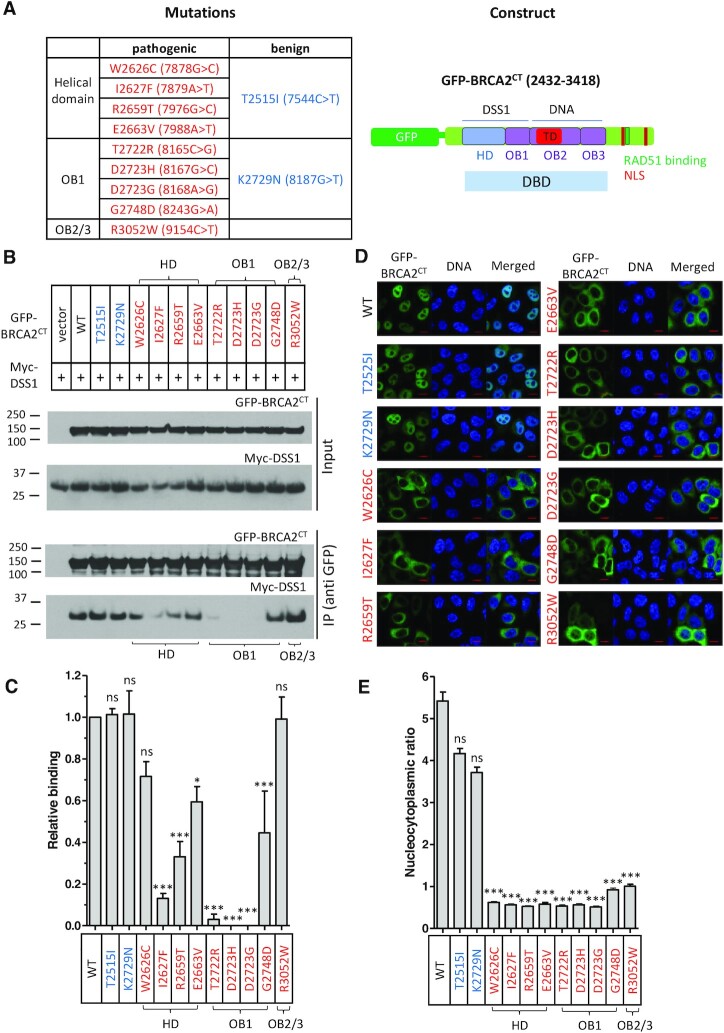
Pathogenic BRCA2 mutations in the DBD affect DSS1 binding and nuclear localization. (**A**) List of pathogenic (red) or benign (blue) mutations in the DBD analysed in this study with a schematic of the expression construct, GFP-BRCA2^CT^. (**B** and **C**) Interaction between the DBD mutants and DSS1. Wild-type or mutant forms of GFP-BRCA2^CT^ were transiently expressed with Myc-DSS1 in 293T cells. Interaction was detected by anti GFP-IP and western blotting with anti-Myc antibody. The graph (**C**) shows DSS1-binding activity of the mutants normalised to the WT value. Binding activity was assessed by quantifying the intensity of the Myc-DSS1 bands pulled-down by IP. Data are presented as mean ± s.e. from three repeats. Statistical significance was assessed by 1-way ANOVA test, followed by Dunnett's multiple comparison test (compared to WT). (D and E) Nucleocytoplasmic distribution of the DBD mutants. HeLa cells transiently expressing GFP-BRCA2^CT^ fragments were fixed with PFA before imaging. The graph (**E**) shows the GFP intensity ratio (nuclear/cytoplasmic, mean ± s.e.). More than 100 cells per sample were analysed. Statistical significance was assessed by the Kruskal-Wallis test, followed by Dunnett's multiple comparison test (compared to WT). A representative result from two independent experiments is shown. (**D**) shows representative microscopic images. Scale bar, 10μm.

The clustering of *BRCA2* pathogenic missense mutations in the HD and OB1 regions of the DBD prompted us to investigate their effect on DSS1 binding. To this end, we expressed in 293T cells constructs encoding green fluorescent protein (GFP) fused to either wild-type (WT) or mutant forms of the carboxyl-terminal one-third of BRCA2 (GFP-BRCA2^CT^), spanning residues 2432 to 3418, encompassing the DBD and NLSs (Figure 2A). As expected, GFP-BRCA2^CT^ WT could be co-immunoprecipitated with co-expressed Myc-epitope tagged DSS1 (Myc-DSS1). Mutant forms of GFP-BRCA2^CT^ encoding the benign variants T2515I (in the HD) or K2729N (in the OB1) also interacted with Myc-DSS1 (Figure 2B, lanes 2, 3 and 4).

In contrast, GFP-BRCA2^CT^ bearing pathogenic mutations exhibited reduced or undetectable binding to Myc-DSS1. Notably, different pathogenic mutants were differentially affected, with the following order of DSS1 binding - D2723H, D2723G (*lowest* DSS1 binding) < T2722R << I2627F < R2659T < G2748D << E2663V < W2626C (*highest* DSS1 binding). The pathogenic W2626C and E2663V mutants were least affected. The remaining pathogenic mutants all showed a marked decrease, with undetectable DSS1 binding in the D2723H and D2723G mutants, which affect a key Asp residue in the OB1-fold, as well as T2722R. All of these residues are evolutionarily conserved, and either in direct contact with DSS1 (R2659 and G2748), or adjacent to DSS1 contacts (W2626, I2627, E2663, T2722, and D2723) in the BRCA2 DBD/DSS1/ssDNA structure (33). In particular, D2723 is involved in hydrogen bonding with other residues in OB1 (S2670, I2672 and W2725), to stabilize the HD / OB1 interface (33,45). Our observations therefore provide direct evidence that residues in the DBD affected by these pathogenic mutations are important for DSS1 binding.

By contrast, however, the pathogenic R3052W mutant, which is located at the OB2/ OB3 junction outside the DSS1-binding region of the DBD, engaged Myc-DSS1 just as effectively as the WT or benign mutant forms (Figure 2B and C). Thus, the pathogenicity of the mutant may not be related to the loss of DSS1 binding.

### Pathogenic BRCA2 mutations in the DBD affect nuclear localization

Whereas WT BRCA2 is normally localized primarily in the cell nucleus, mutant BRCA2 carrying the pathogenic mutation D2723H affecting Asp^2723^ has previously been reported to be mis-localized to the cytosol (46,47). Moreover, we have previously shown that the cytoplasmic mis-localization of BRCA2^D2723H^ is accompanied by the loss of DSS1 binding (36). Accordingly, we tested the subcellular localization of WT or mutant forms of GFP-BRCA2^CT^ by expressing them in HeLa cells and measuring nuclear / cytoplasmic ratio of GFP intensity from the microscopic images (Figure 2D and E).

Like WT GFP-BRCA2^CT^, the benign mutants T2515I and K2729N were primarily nuclear localized (nuclear / cytoplasmic ratio >3). In contrast, all forms of GFP-BRCA2^CT^ bearing pathogenic mutations localized primarily to the cytosol (nuclear / cytoplasmic ratio ≤1). Reduced nuclear localization was particularly marked in the D2723H mutant, whereas three other pathogenic mutants showed statistically significant higher ratios of nuclear / cytoplasmic localization compared to D2723H (Kruskal-Wallis test followed by Dunn's multiple comparison Test, W2626C: *, G2748D: ***, R3052W: ***). Interestingly, even those mutants with substantial Myc-DSS1-binding activity (W2626C, E2663V and G2748D; Figure 2B and C) showed significantly reduced nuclear localization. Thus, our results suggest that even a small reduction in DSS1 binding significantly impairs the nuclear localization of cancer-causing *BRCA2* mutants within the DSS1-binding region of the DBD.

On the other hand, the pathogenic R3052W mutant, located outside the DSS1-binding region of the DBD, also showed significantly reduced nuclear localization despite having similar DSS1 binding activity to that of the WT form (Figure 2B and C). Again, this result suggests that the pathogenicity of this mutant is unrelated to its DSS1-binding capacity.

### Computational modelling of mutant BRCA2 DBD folding

Calculation of the change in chemical energy of a protein structure upon mutation (Gibbs free energy, ΔΔG) can be used to predict the influence of a mutation on the folded state of the protein (48,49). ΔΔG of a high enough energy provides evidence for an unfolding of the protein or protein surface, leading to mutational inactivation or improper protein trafficking and expression, especially if the mutation is buried within the protein structure.

To investigate the effect of BRCA2 DBD mutations on protein folding, we computationally modelled their consequences upon incorporation into the three available crystal structures (PDB reference 1MIU, 1MJE, 1IYJ) of rodent Brca2 DBD/Dss1 complexes (33). We exclusively studied pathogenic mutations affecting evolutionarily conserved residues (W2626C, I2627F, R2659T, E2663V, T2722R, D2723H, D2723G, G2748D and R3052W), since the amino acid sequence of rodent Brca2 DBD is 56% homologous to the human. We compared them to benign variants (K2729N, R2842H, R2888C, V2969M, R2973C, V3079I, D3170G) also affecting conserved amino acids. Our analyses (see Materials and Methods) point to two major effects that distinguish pathogenic DBD mutations from benign variants.

First, we calculated the change in Gibbs free energy (ΔΔ*G*) (50,51) upon mutation in all three crystal structures. A positive ΔΔG marks a destabilizing mutation, with a ΔΔ*G* ≥2.5 kcal/mol considered highly likely to destabilize the protein structure (49,52,53). We infer from these analyses that the D2723H mutation induces a positive ΔΔ*G* ≥10 kcal/mol in all three crystal structures (Supplementary Figure S1A), predicting that it destabilizes DBD folding. In addition, six other pathogenic mutations (W2626C, I2627F, T2722R, D2723G, G2748G and R3052W) are predicted to induce a ΔΔ*G* ≥2.5 kcal/mol in at least two of the three crystal structures, suggesting that they also destabilize DBD folding.

A second analysis provides additional evidence for the effects of pathogenic DBD mutations on protein folding. Relative solvent accessibility (RSA) (54) measures how buried an amino acid is relative to its sidechain surface area, with residues that are completely buried (defined as those with an RSA ≤0.2) more likely to destabilize protein structure and cause unfolding (55). All pathogenic mutations are found to be completely buried within the protein structure, and whilst the benign mutations show a range of RSA values, the most buried benign mutations (V2969M and V3079I) show stabilizing or negligible changes in ΔΔ*G*.

The rank order of calculated changes in ΔΔG or RSA were not directly correlated with the pathogenicity of BRCA2 DBD mutants. For example, the pathogenic mutant R2659T exhibits only a modest ΔΔG, whereas the benign R2842H mutant exhibits a higher ΔΔG than certain pathogenic mutants (Supplementary Figure S1A). However, when comparing the average ΔΔ*G* and RSA of the mutants across all structures studied, we find that pathogenic and benign mutations are significantly different (independent *t*-test *P* < 0.001 in both cases) (Supplementary Figure S1B), with pathogenic mutations exhibiting significantly higher average energy of mutation, and lower RSA. These findings are similar to those in a recent study of NOTCH1 and TP53 mutation selection in human skin (53). Taken together, our findings raise the possibility that pathogenic DBD mutants impair BRCA2 function by affecting protein folding.

### The BRCA2–DSS1 interaction antagonizes intracellular BRCA2 oligomerization

Brh2, the BRCA2 ortholog in the fungus *Ustilago maydis*, has been shown to self-associate through its DSS1-binding domain to form oligomers, the dissociation of which via DSS1 interaction has been suggested as a regulatory mechanism for Brh2 function (56). To investigate whether human BRCA2 is regulated in a similar way, we first tested whether human BRCA2 self-interacts via the carboxyl-terminal DSS-binding regions encoded in the BRCA2^CT^ fragment spanning residues 2432–3418.

**Figure 3.**
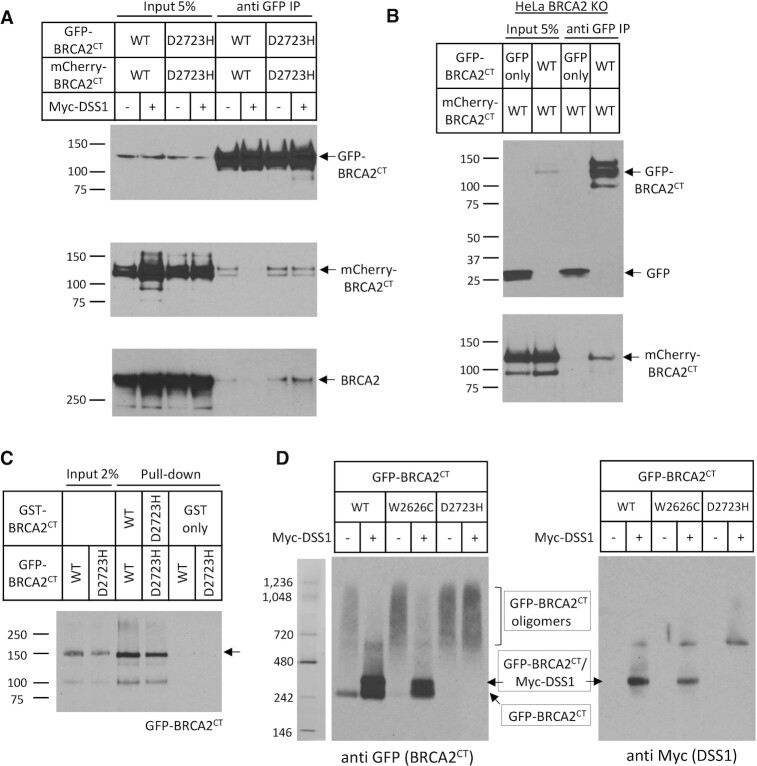
The BRCA2-DSS1 interaction antagonizes intracellular BRCA2 oligomerization. (**A**) Self-oligomerization of BRCA2^CT^ is suppressed by Myc-DSS1. Wildtype or D2723H mutant forms of BRCA2^CT^, tagged with either GFP or mCherry, were transiently expressed in 293T cells with or without Myc-DSS1. Interaction was detected by anti GFP-IP and western blotting for mCherry-BRCA2^CT^ and endogenous BRCA2. (**B**) Self-oligomerization of BRCA2^CT^ fragments in the BRCA2 Knock-out (KO) HeLa cells. BRCA2^CT^ fragments tagged with either GFP or mCherry were co-expressed in BRCA2 KO HeLa cells. Their interaction was detected by anti GFP-IP and western blotting for mCherry-BRCA2^CT^. GFP vector alone serves as a negative control. (**C**) GST-pull down assay. Purified GST-BRCA2^CT^ fragments bound to the Glutathione Sepharose 4B beads were incubated with purified GFP-BRCA2^CT^ fragments. The beads were washed, and bound proteins were eluted, before separation by SDS-PAGE. Interactions were detected by western blotting for GFP-BRCA2^CT^. GST alone serves as a negative control. (**D**) Native gel electrophoresis of GFP-BRCA2^CT^. Extracts from HeLa cells transiently expressing GFP-BRCA2^CT^ forms with or without Myc-DSS1 were analysed by native gel electrophoresis. The bands corresponding to GFP-BRCA2^CT^ oligomers, GFP-BRCA2^CT^/Myc-DSS1 complex and GFP-BRCA2^CT^ are marked with arrows.

To this end, we co-expressed in 293T cells BRCA2^CT^ fragments tagged either with GFP (GFP-BRCA2^CT^) or mCherry (mCherry-BRCA2^CT^). Both WT and D2723H mutant forms of GFP-BRCA2^CT^ pulled-down their mCherry tagged counterparts (Figure 3A, mCherry western). Moreover, both WT and D2723H GFP-BRCA2^CT^ fragments interact with endogenous BRCA2 protein in transfected cells (Figure 3A, lower panel, BRCA2 western). Notably, an interaction between the BRCA2 N-terminal (1–714) and C-terminal (2300–3418) regions, which is also disrupted by DSS1, has been recently detected in an *in vitro* analysis using purified protein fragments (57).

We therefore sought to test whether the observed self-interaction between the BRCA2^CT^ fragments might be bridged via an N-terminal-to-C-terminal interaction with the full-length endogenous BRCA2 protein. To this end, we generated a *BRCA2* knock-out HeLa cell line (BRCA2 KO, Supplementary Figure S2A) by CRISPR/Cas9 gene editing, and then co-expressed GFP-BRCA2^CT^ and mCherry-BRCA2^CT^ in this cell line. Despite the absence of full-length endogenous BRCA2 protein, GFP-BRCA2^CT^ retains its interaction with mCherry-BRCA2^CT^ (Figure 3B). These findings suggest that the observed self-interaction between the BRCA2^CT^ fragments is not bridged by the endogenous BRCA2 protein. We further corroborated this result by testing whether there was an interaction between BRCA2^CT^ fragments tagged either with GST (GST-BRCA2^CT^) or GFP (GFP-BRCA2^CT^) purified from *E. coli* (Supplementary Figure S2B). Both WT and D2723H mutant forms of purified GST-BRCA2^CT^ fragments exhibited an interaction with their cognate GFP-tagged forms (Figure 3C). Collectively, our findings suggest that the self-interaction we observe between BRCA2^CT^ fragments is direct, and distinct from the BRCA2 N-terminal-to-C-terminal interaction that was reported recently (57).

Interestingly, expression of Myc-DSS1 disrupts the interaction between GFP- and mCherry-tagged WT BRCA2^CT^ fragments, but not the interaction between GFP- and mCherry-tagged D2723H mutant BRCA2^CT^ fragments (Figure 3A, mCherry western). The interaction of endogenous BRCA2 with WT GFP-BRCA2^CT^ is also disrupted by Myc-DSS1, but not its interaction with D2723H mutant GFP-BRCA2^CT^ (Figure 3A, lower panel, BRCA2 western). These results suggest firstly, that wildtype BRCA2 can self-interact via the carboxyl-terminal DSS1-binding regions encoded in the BRCA2^CT^ fragment spanning residues 2432–3418, and that this interaction is suppressed by DSS1 binding. Second, they suggest that the D2723H mutation, which markedly suppresses DSS1 binding, leads to persistent self-oligomerization that is refractory to DSS1 expression.

These conclusions are further supported by evidence from native gel electrophoresis of intracellular forms of wildtype versus mutant GFP-BRCA2^CT^ fragments expressed, when Western blotted with anti-GFP. In cell extracts, wildtype GFP-BRCA2^CT^ migrates in two forms – first, a faster-migrating species consistent with a monomeric form, and second, as a smear of species with retarded gel mobility that may represent oligomeric forms. Co-expression of Myc-DSS1 alters this pattern. The smear of slow-migrating forms of GFP-BRCA2^CT^ is focused into one prominent band, which migrates more slowly than the band corresponding to a GFP-BRCA2^CT^ monomer (Figure 3D, left panel). Western blotting with anti-Myc antibodies shows that the faster-migrating of these two bands does not contain Myc-DSS1 (Figure 3D, right panel), suggesting that it represents monomeric GFP-BRCA2^CT^ alone or in complex with other proteins. By contrast, the slower-migrating band (Figure 3D, right panel) contains Myc-DSS1, consistent with a monomeric Myc-DSS1/GFP-BRCA2^CT^ complex.

Myc-DSS1 expression has varied effects on the native gel mobility of two different mutant GFP-BRCA2^CT^ fragments. The W2626C mutant (which retains residual DSS1 binding activity (Figure 2B and C)) migrates predominantly as apparent oligomers. Like the wildtype GFP-BRCA2^CT^ protein, Myc-DSS1 co-expression focuses the W2626C mutant into one band consistent with a monomeric GFP-BRCA2^CT^ W2626C / Myc-DSS1 complex (besides a second band consistent with monomeric GFP-BRCA2^CT^ W2626C alone or in complex with other proteins) as described above (Figure 3D). In contrast, the D2723H mutant (which is devoid of detectable DSS1 binding activity) persistently migrates as apparent oligomers even when co-expressed with Myc-DSS1 (Figure 3D).

Thus, these results suggest that mutant BRCA2^CT^ expressed in cells form self-oligomers to a greater extent than wildtype forms. Overexpressed DSS1 antagonizes oligomer formation by wildtype BRCA2^CT^, or its pathogenic W2626C mutant which retains residual DSS1-binding activity, but not the D2723H mutant form.

### Pathogenic DBD mutants introduced into the endogenous *BRCA2* gene cause cytosolic mis-localization and disable DNA repair by HDR

We used two pathogenic BRCA2 DBD mutants—W2626C and D2723H, affecting the HD and OB1-fold respectively—to exemplify effects on the function of BRCA2 in HDR. To this end, we used CRISPR/Cas9 gene editing to generate HeLa Kyoto cells bearing homozygous full-length forms of each of these BRCA2 mutations (BRCA2^W2626C/W2626C^ or BRCA2^D2723H/ D2723H^; hereafter called HeLa-W2626C or HeLa-D2723H). Two homozygous clones for each mutation validated by nucleotide sequencing were used for further studies.

**Figure 4.**
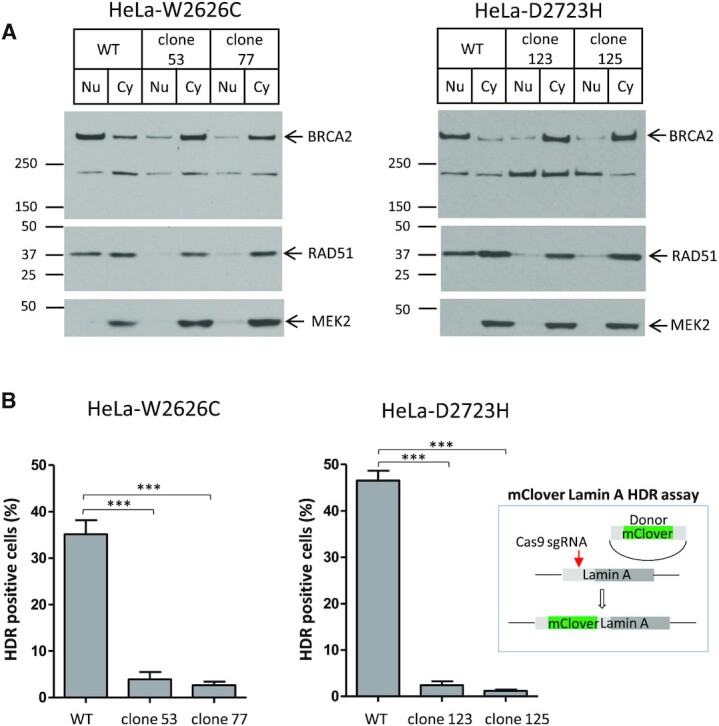
Pathogenic DBD mutants introduced into the endogenous *BRCA2* gene cause cytosolic mis-localization and disable DNA repair by HDR. (**A**) Subcellular fractionation of *BRCA2* mutant cell lines, HeLa-W2626C and HeLa-D2723H. Two independent clones for each mutation were used. Nuclear and cytoplasmic fractions of BRCA2 and RAD51 were analysed by western blotting. MEK2 is a cytoplasmic marker. (**B**) mClover Lamin A HDR assay. Cells transfected with Lamin A-targeting sgRNA and mClover Lamin A donor constructs as shown in the schematic were analysed for mClover Lamin A-positive cells. Mean of HDR positive cells (%) ± s.e. from two repeats is shown. More than 100 cells per sample were analysed in each repeat. Statistical significance was tested by one-way ANOVA test, followed by Bonferroni's multiple comparison test.

We first tested the intracellular localization of the BRCA2^W2626C^ and BRCA2^D2723H^ mutants by fractionation into nuclear and cytosolic fractions, which were analysed by western blotting. Consistent with our results for the GFP-BRCA2^CT^ fragments, both BRCA2^W2626C^ and BRCA2^D2723H^ mutants localized primarily in the cytosol, as was RAD51, suggesting that mutant BRCA2/RAD51 complexes were mis-localized in the mutant cell lines (Figure 4A).

Next, we measured the proficiency of HeLa-W2626C or HeLa-D2723H in carrying out HDR using the mClover Lamin A assay (40,41). Cells were transfected with the construct expressing Cas9/*Lamin A* targeting sgRNA and the donor construct consisting of mClover sequence flanked by *Lamin A* homology arms. Three days after transfection, cells were fixed and analysed by microscopy. Close to 40% of WT cells were mClover Lamin A positive reflecting faithful incorporation of mClover sequence into *Lamin A* gene by HDR. In contrast, both mutants showed very low HR efficiency (Figure 4B and Supplementary Figure S3).

Thus, we show using two different DBD mutants introduced into the endogenous *BRCA2* gene by CRISPR/Cas9 mutagenesis that the disruption of DSS1 binding causes cytosolic mis-localization of the encoded BRCA2 proteins, and disables DNA repair by HDR. Interestingly, the W2626C or D2723H mutations affect BRCA2 localization and impair HDR to similar degrees, even though the BRCA2 W2626C mutant retains residual DSS1-binding capacity whereas the D2723H mutant does not. Our observations speak to the importance of DSS1 engagement for the transport of BRCA2 to its sites of activity in the cell nucleus.

### Pathogenic DBD mutants exhibit defective nuclear import

**Figure 5.**
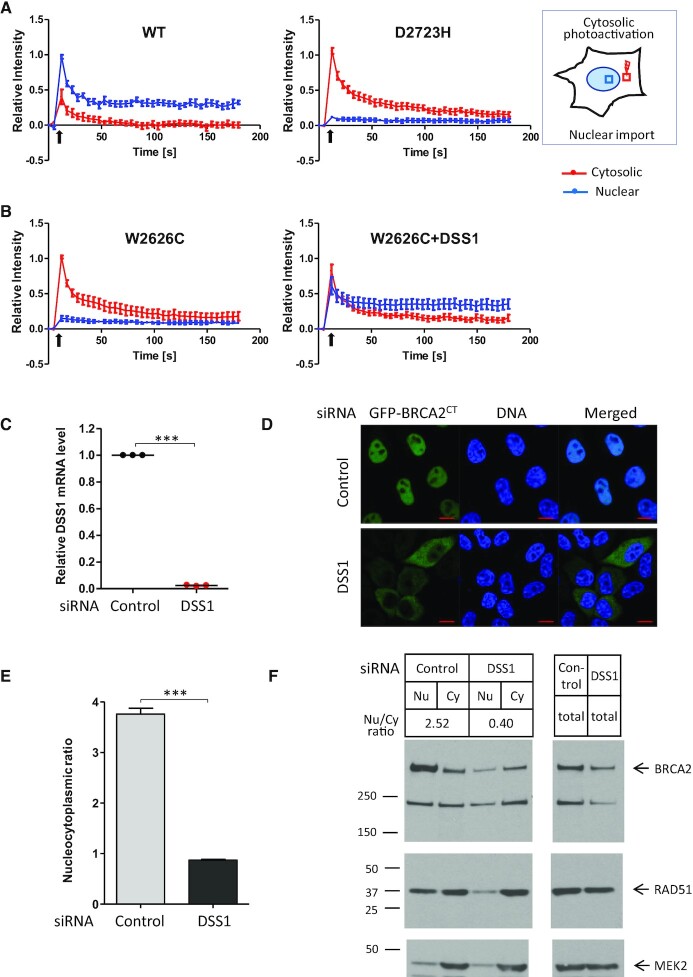
Pathogenic DBD mutants exhibit defective nuclear import. (**A**) Nuclear import assay using BRCA2^CT^ tagged with photoactivable GFP (paGFP). HeLa cells expressing wildtype or mutant forms of paGFP-BRCA2^CT^ were monitored by live imaging. As shown in the schematic, photoactivation of a cytosolic area (red square) was followed by measurement of fluorescence intensity in the designated area in the cytosol (red square) and the nucleus (blue square). Plots show the relative fluorescence intensity (mean ± s.e.), normalized to the maximum intensity in each cell. 8–10 cells per sample were analysed. Black arrows mark the timing of photoactivation. (**B**) HeLa cells expressing paGFP-BRCA2^CT^ W2626C with or without Myc-DSS1 were monitored by live imaging after cytosolic photoactivation and analysed as shown in (A). (**C**) Efficiency of DSS1 depletion by siRNA transfection. Relative level of DSS1 mRNA measured by RT-qPCR assay, a result from technical triplicate is shown. Statistical significance was assessed by paired *t*-test. (**D** and **E**) Nucleocytoplasmic distribution of the GFP-BRCA2^CT^ fragment after DSS1 depletion. HeLa cells were transfected with control or DSS1 siRNA 24 h before transfecting with the GFP-BRCA2^CT^ construct. Nucleocytoplasmic distribution was analysed as described in Figure 2E. The graph (E) shows the GFP intensity ratio (nuclear/cytoplasmic, mean ± s.e.). More than 100 cells per sample were analysed. Statistical significance was assessed by unpaired t-test. A representative result from three independent experiments is shown. Representative microscopic images are shown in (D). Scale bar, 10 μm. (**F**) Subcellular fractionation of HeLa cells after DSS1 depletion. HeLa cells were transfected with control or DSS1 siRNA 48 h before fractionation. Nuclear and cytoplasmic fractions of BRCA2 and RAD51 were analysed by western blotting. The nuclear/cytoplasmic ratio of BRCA2 expression is shown. MEK2 is a cytoplasmic marker. Total cell extracts are also shown. A representative result from two independent experiments is shown.

To investigate what causes pathogenic BRCA2 DBD mutants to mis-localize in the cytosol, we employed a photoactivable GFP (paGFP) assay (58,59) to test their nuclear import. We expressed paGFP-tagged wildtype or mutant BRCA2^CT^ fragments in HeLa Kyoto cells, before photoactivating a defined cytoplasmic region using a 405 nm laser. The migration of photoactivated species from the cytosol to the nucleus was then monitored over a 3-minute period (Figure 5A, experimental scheme).

Wildtype paGFP-BRCA2^CT^ exhibited low levels of cytosolic photoactivation, consistent with the predominantly nuclear localization of wildtype BRCA2. Photoactivated wildtype paGFP-BRCA2^CT^ was rapidly transported into the cell nucleus, as indicated by a rapid increase in nuclear fluorescence. This nuclear fluorescence decreased over time due to diffusion within nucleus until equilibrium was reached at 1 min after photoactivation. At equilibrium, nuclear fluorescence was maintained at ∼30% of the initial peak, whereas cytosolic fluorescence was at basal levels, indicative of the efficient nuclear import of BRCA2^CT^.

In contrast to wildtype, the paGFP-BRCA2^CT^ D2723H mutant exhibited a high level of cytosolic photoactivation consistent with its concentration in the cytosol. After photoactivation, there was no detectable increase in nuclear fluorescence (although cytosolic fluorescence intensity dissipated from the photoactivated region due to diffusion). When equilibrium was reached at 3 min, there was only a minimal increase in nuclear intensity, speaking to a defect in nuclear import (Figure 5A and Supplementary Figure S4). Collectively, these results suggest that the paGFP-BRCA2^CT^ D2723H mutant protein is not efficiently transported to the nucleus.

### DSS1 expression restores the nuclear localization of pathogenic BRCA2 DBD mutants

We find (Figure 3) that both wildtype and mutant forms of the BRCA2^CT^ protein form self-oligomers in cell extracts, and that DSS1 expression can antagonize oligomerization of the wildtype but not mutant forms. These observations prompted us to test whether DSS1 expression could also affect the nuclear localization of pathogenic BRCA2 DBD mutant proteins.

**Figure 6.**
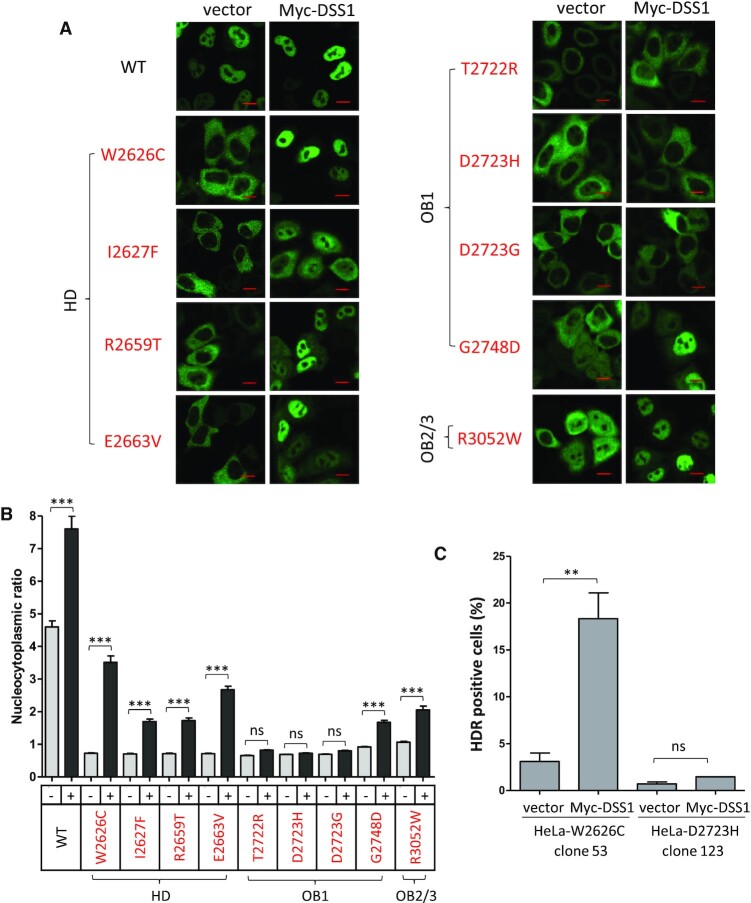
DSS1 expression restores the nuclear localization and HDR function of hypomorphic BRCA2 pathogenic mutants. (**A** and **B**) Nucleocytoplasmic distribution of the DBD mutants with DSS1 overexpression. HeLa cells expressing GFP-BRCA2^CT^ forms without (vector only) or with Myc-DSS1 were fixed with PFA and analysed by microscopy. Representative images are shown. Scale bar, 10μm. The graph (B) shows GFP intensity ratio (nuclear/cytoplasmic, mean ± s.e.) measured from the images. Myc-DSS1 expression increases the nuclear/cytoplasmic ratio of GFP-BRCA2^CT^ fragments to varying degrees: WT by 1.7 fold; W2626C and E2663V mutants by 3.5- to 5-fold; I2627F, R2659T, and G2748D mutants by 1.8- to 2.4-fold; T2722R, D2723H, and D2723G mutants, little change. More than 100 cells per sample were analysed. Statistical significance was assessed by one-way ANOVA test, followed by Bonferroni's multiple comparison test. A representative result from three independent experiments is shown. (**C**) mClover Lamin A HDR assay. HeLa cells harbouring BRCA2 mutations (Figure 4) were transfected with vector or Myc-DSS1 plasmids in addition to mClover Lamin A HDR assay plasmids. Mean of HDR positive cells (%) ± s.e. from two repeats is shown. More than 100 cells per sample were analysed in each repeat. Statistical significance was assessed by one-way ANOVA test, followed by Bonferroni's multiple comparison test.

Accordingly, we co-expressed the wildtype or different mutant forms of GFP-BRCA2^CT^ with Myc-DSS1, and compared their nuclear / cytoplasmic distribution by microscopy (Figure 6A and B). Myc-DSS1 expression modestly increased the nuclear/cytoplasmic ratio of wildtype GFP-BRCA2^CT^ by 1.7-fold. Myc-DSS1 expression had a similar effect on the distribution of the mutant proteins but to varying degrees. Whereas the nuclear / cytoplasmic ratio of W2626C and E2663V mutants increased by 3.5- to 5-fold, and that of the I2627F, R2659T and G2748D mutants by 1.8- to 2.4-fold, the T2722R, D2723H and D2723G mutants showed little change in nuclear localization. Thus, our findings collectively speak to the idea that DSS1 antagonizes the self-oligomerization of BRCA2 DBD to promote its nuclear localization.

Notably, the rank order in which DSS1 expression restored the nuclear localization of different BRCA2 DBD mutant proteins was similar to the rank order of DSS1 binding by the mutant proteins (Figure 2C), but did not follow a simple linear relationship. Nevertheless, the nuclear localization of mutants that retain residual DSS1-binding was corrected by DSS1 overexpression, whereas the D2723H and D2723G mutants which severely lack DSS1 binding was not. One possibility raised by these findings is that a critical threshold of DSS1 binding may be required for BRCA2 transport and function.

To further substantiate and extend this conclusion, we also tested the effect of Myc-DSS1 expression on the nuclear import of the paGFP-BRCA2^CT^ W2626C mutant protein using the paGFP assay. The GFP-BRCA2^CT^ W2626C mutant exhibits reduced but not absent DSS1 binding (Figure 2C), and its oligomerization can be suppressed by Myc-DSS1 expression (Figure 3D). The nuclear import of the paGFP-BRCA2^CT^ W2626C mutant was impaired to a similar degree (Figure 5B, left panel) as the D2723H mutant. Strikingly, Myc-DSS1 co-expression markedly increases the nuclear import of paGFP-BRCA2^CT^ W2626C (Figure 5B, right panel), restoring it to a level akin to the wildtype paGFP-BRCA2^CT^ protein.

We find that over-expressed DSS1, but not its endogenous levels, can restore the nuclear localization of certain mutant forms of BRCA2. This finding raises the possibility that endogenous DSS1 is rate-limiting for BRCA2 nuclear transport. Accordingly, we depleted DSS1 from HeLa cells by siRNA transfection (Figure 5C) before expressing GFP-BRCA2^CT^. The nucleocytoplasmic ratio of GFP-BRCA2^CT^ was significantly decreased to an extent similar to that of the mutant forms (Figure 5D and E). Notably, subcellular fractionation followed by western blotting also shows that DSS1 depletion in HeLa cells reduces the nuclear localization of full-length endogenous BRCA2 (Figure 5F). We note that total level of BRCA2 decreases after DSS1 depletion, consistent with a previous report (35). These results together provide further lines of evidence that DSS1 binding by the BRCA2 DBD is essential for the nuclear localization of BRCA2.

Interestingly, we find that the GFP-BRCA2^CT^ R3052W mutant (which retains its DSS1-binding ability (Figure 2C)) is also mis-localized to the cytosol, and that its nuclear localization is increased by 1.9-fold through Myc-DSS1 expression (Figure 6B). The R3052W mutation is predicted to disrupt a hydrogen bonding network that supports an interaction between OB2 and OB3 domains, outside DSS1 interaction sites (45,46), and therefore probably affects BRCA2 DBD folding. In addition, the nuclear localization of GFP-BRCA2^CT^ wildtype protein is also slightly increased by Myc-DSS1, consistent with our observation that wildtype GFP-BRCA2^CT^ protein exhibits a small degree of self-oligomerization antagonized by DSS1 (Figure 3D).

Thus, when considered together, our findings provide multiple lines of evidence to support a model wherein the regulated assembly of BRCA2/DSS1 monomers from BRCA2 oligomers assists in the normal folding, assembly and nuclear transport of BRCA2. Cancer-causing mutations in the DBD, either by diminishing DSS1 binding and/or by affecting DBD folding, perturb this intracellular assembly mechanism to impair nuclear localization. Notably, effects on DBD folding may in turn also impair the DNA binding ability of these mutants.

### Expression of DSS1 restores HDR in cells bearing hypomorphic BRCA2 pathogenic mutants

Our observation that increased DSS1 expression enhances the nuclear localization of BRCA2 DBD mutant proteins prompted us to test for the restoration of DNA repair by HDR. To this end, we used the mutant cell lines HeLa-W2626C or HeLa-D2723H (Figure 4), in which CRISPR/Cas9 engineering was used to introduce either the W2626C or D2723H mutation into both endogenous alleles of *BRCA2*. Myc-DSS1 expression in HeLa-W2626C significantly increased HDR measured by the mClover Lamin A assay, by 6-fold from 3% to 18% (roughly half the level of wildtype cells). By contrast, Myc-DSS1 expression did not significantly affect HDR in HeLa-D2723H cells (Figure 6C). Thus, the HDR defect in cells bearing the pathogenic W2626C mutation in *BRCA2* – a hypomorphic variant that exhibits reduced but not absent DSS1 binding - can be corrected by DSS1 expression. However, cells carrying the pathogenic D2723H mutant devoid of detectable DSS1 binding could not be corrected in this way. Together, these results provide further evidence that cancer-causing BRCA2 missense mutations disrupt a DSS1-mediated intracellular protein assembly mechanism essential for its function in DNA repair by HDR, and suggest an approach for therapeutic correction via drugs that mimic the effects of DSS1 binding.

## DISCUSSION

We identify here a common mechanism underlying the pathogenicity of 9 out of 13 missense mutations in human BRCA2 classified as cancer-causing by expert panel review from amongst 37 known mis-sense mutations. The mutations we have analysed cluster in the short, ∼340 residue DBD of human BRCA2, which represents only ∼10% of the 3418 residue BRCA2 protein. Four additional missense mutations also affect conserved residues in the same short BRCA2 segment, and may therefore act by a similar mechanism, although their pathogenicity was confirmed by expert panel review only recently (13) during the completion of the studies reported here. We show that each of the pathogenic mutations we have analysed mis-localizes BRCA2 to the cytosol, extending previously reported findings that the D2723H DBD mutation acts so (36,46,47). We demonstrate that pathogenic mis-sense mutations in the HD, OB1 or OB2 regions of the DBD inhibit, to varying degrees, DSS1 binding by BRCA2. Moreover, we provide evidence that the introduction into endogenous *BRCA2* of mis-sense mutations that either retain residual DSS1-binding capacity (the W2626C mutant), or ablate it more severely (the D2723H mutant) have similar effects in impairing the nuclear localization of full-length BRCA2 protein, as well as its essential function in DNA repair by HDR. Our results have important implications not only because inherited mutations affecting *BRCA2* are amongst the commonest causes of human cancer susceptibility, but also because deciphering their effects on BRCA2 function is essential to assess cancer risk, and devise new approaches for treatment or prevention.

We provide evidence that intracellular BRCA2 undergoes self-oligomerisation that is regulated by DSS1, reminiscent of elegant genetic studies in *U. maydis* that first identified such a process affecting the orthologous protein, Brh2 (56). Our results extend and complement recently reported *in vitro* biochemical studies which show that purified BRCA2 protein self-associates via amino (N)- carboxyl (C) terminal and N-N terminal interactions, and that the N-C terminal self-interaction is sensitive to inhibition by DSS1 and single-stranded (ss)DNA (57). We provide here evidence that self-interaction can also directly occur between the C-terminal regions of BRCA2, and that this C-C terminal interaction contributes to the intracellular oligomerization of BRCA2 in a manner that can be antagonized by DSS1 overexpression. Interestingly, we find that the intracellular oligomerization and cytosolic mis-localization of pathogenic BRCA2 DBD mutations that diminish but do not ablate DSS1 binding is suppressed by DSS1 overexpression, whereas the oligomerization of DBD mutants devoid of detectable DSS1 binding is unaffected. Thus, our studies suggest that the disruption of DSS1-regulated BRCA2 assembly and nuclear transport underlies the pathogenicity of cancer-causing mutations affecting the DBD.

However, the mechanisms by which DSS1 regulates BRCA2 function via its interaction with the DBD are likely to be complex, because pathogenic BRCA2 DBD mutations consistently impair the intracellular assembly and nuclear localization of BRCA2 in a manner that is not directly related to their DSS1-binding capacity. For example, the W2626C mutant (which retains significant DSS1 binding) forms intracellular oligomers and is as impaired in its nuclear localization and loss of HDR function as the D2723H mutant (which lacks detectable DSS1 binding). These findings suggest that even small decreases in DSS1 binding capacity may impair BRCA2 transport and function, again speaking to the possibility that there is a critical threshold of DSS1 binding below which mutant BRCA2 molecules are rendered defective.

**Figure 7.**
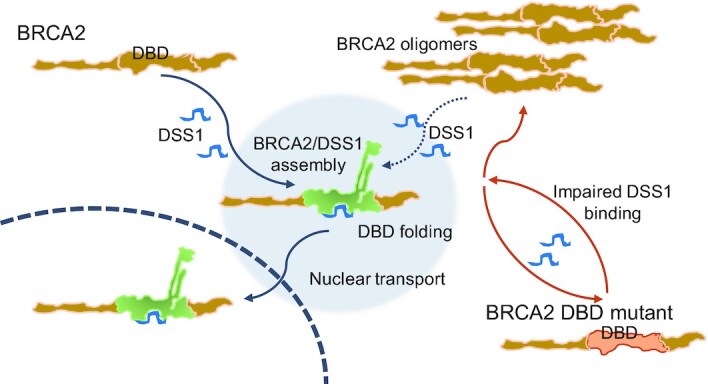
Cancer-causing BRCA2 missense mutations disrupt an intracellular protein assembly mechanism to disable genome maintenance. Our results suggest a hypothetical model wherein the engagement of DSS1 by the DBD of BRCA2 antagonizes BRCA2 self-oligomerization and promotes protein folding. Assembly of a correctly folded BRCA2/DSS1 complex enables nuclear transport. Cancer-causing mutations in the BRCA2 DBD impair DSS1 binding, perturbing this protein assembly mechanism, and provoking the formation of intracellular BRCA2 oligomers that are excluded from the cell nucleus. The failure to assemble correctly folded mutant BRCA2/DSS1 complexes, and cytosolic mis-localization of the mutant BRCA2, disables HDR in cells harbouring the DBD mutations. DSS1 can antagonize the self-oligomerization of wild-type BRCA2, and of cancer-causing DBD mutants such as W2626C that retain some degree of DSS1 binding.

Taken together, these findings suggest a hypothetical model (Figure 7) wherein DSS1 regulates the intracellular oligomerization of BRCA2 in a protein assembly mechanism that is essential for nuclear transport, consistent with its proposed activity as a chaperone that assists the folding and intracellular trafficking of multi-protein complexes (60). Indeed, we show here using photoactivation and dynamic fluorescence imaging that defects in nuclear transport contribute to the mis-localization of multiple pathogenic BRCA2 DBD mutants, beyond the effects of DSS1 binding on nuclear export of the D2723H mutant that we previously reported (36). However, pathogenic BRCA2 mis-sense mutations in the DBD exhibit apparently stereotypic defects in nuclear localization regardless of the degree of defective DSS1-binding. Conversely, BRCA2 D2723H, the pathogenic DBD mutant with the most severe lack of DSS1 binding activity, is still transported to the nucleus albeit in reduced amounts. We therefore suggest that defects in folding, nuclear transport and nuclear export induced by loss of DSS1 binding may collectively contribute to the observed phenotypic defects.

That the localization and function of BRCA2, a large protein essential for genome integrity in dividing human cells, should depend so critically on its interaction with the 70 amino acid DSS1 polypeptide is difficult to reconcile with the robustness typical of biological systems. However, even BRCA2 D2723H, which lacks detectable DSS1 binding, localizes to the nucleus at least in small amounts sufficient for cell viability and growth. Moreover, even the low concentrations at which endogenously tagged BRCA2 protein (eg., (61,62)) is expressed do not appear to be rate-limiting for genome stability in wild-type cells. We speculate that these features may have precluded the selection during evolution of more robust mechanisms for BRCA2 protein folding and intracellular trafficking.

Notably, we find that the impaired nuclear localization and DNA repair function of certain pathogenic BRCA2 DBD mutants that retain DSS1 binding capacity, like W2626C, can be ameliorated by DSS1 overexpression. Since DSS1 encodes a short, 70 residue polypeptide whose binding sites within the BRCA2 DBD have been structurally characterized (33), our results suggest that peptidomimetic agents which engage the DSS1-binding site in certain mutant forms of BRCA2 could correct the functional defect. Thus, our work opens opportunities for cancer prevention in patients who carry particular *BRCA2* missense mutations.

## Supplementary Material

gkab308_Supplemental_File

## Notes

Present address: David Shorthouse & Benjamin A. Hall, Department of Medical Physics and Biomedical Engineering, Malet Place Engineering Building, University College London, Gower Street, London WC1E 6BT, UK.
