# Supplementary material for: Cancer-causing BRCA2 missense mutations disrupt an intracellular protein assembly mechanism to disable genome maintenance
Source: Nucleic Acids Res. 2021 May 12;49(10):5588–604. doi: 10.1093/nar/gkab308 (PMC8191791; doi:10.1093/nar/gkab308)
Supplement: gkab308_Supplemental_File [file gkab308_supplemental_file.pdf]

Cancer-causing *BRCA2* missense mutations disrupt an intracellular protein assembly mechanism to disable genome maintenance

Miyoung Lee, David Shorthouse, Robert Mahen, Benjamin A. Hall and Ashok R. Venkitaraman

Supplementary Data

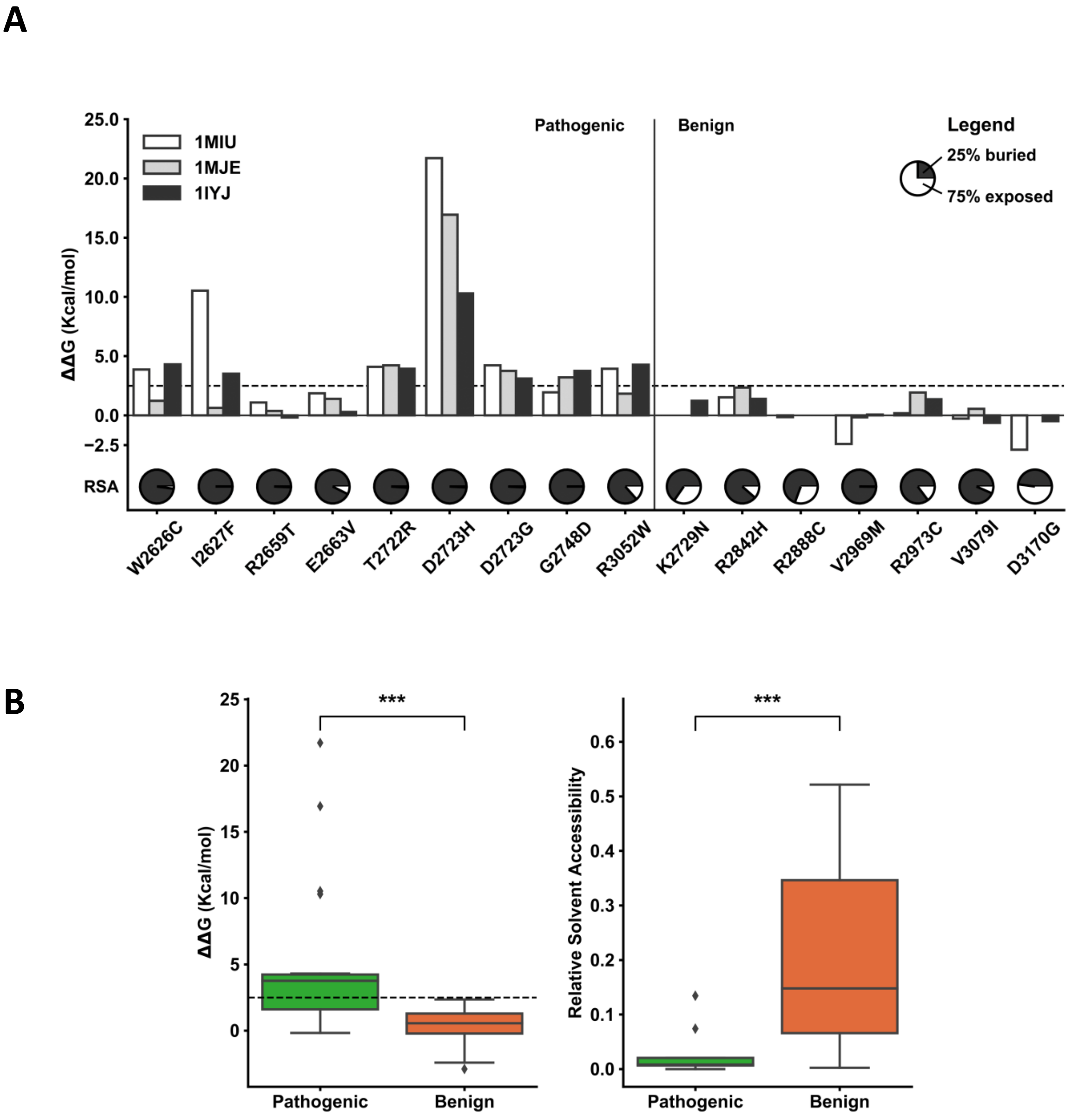

Supplementary Figure 1. Mutational free energy change and relative solvent accessibility (RSA) of BRCA2 DBD mutants. (**A**) Predicted energy of mutation for BRCA2: ΔΔG in Kcal/mol for pathogenic and benign mutations in three structures of rodent Brca2 DBD / DSS1; 1MIU, 1MJE, and 1IYJ. Dotted line represents 2.5 Kcal/mol cutoff. Pie charts represent relative solvent accessibility (RSA) for each residue. Black area indicates proportion of the residue buried compared to a fully solvent exposed same-side chain amino acid. (**B**) Comparison of benign vs pathogenic mutations in three structures of rodent Brca2; 1MIU, 1MJE, and 1IYJ. Left: Average ΔΔG in Kcal/mol. Line represents 2.5 kcal/mol. Right: Relative Solvent Accessibility (RSA). *** represents independent t-test $p \leq 0.001$.

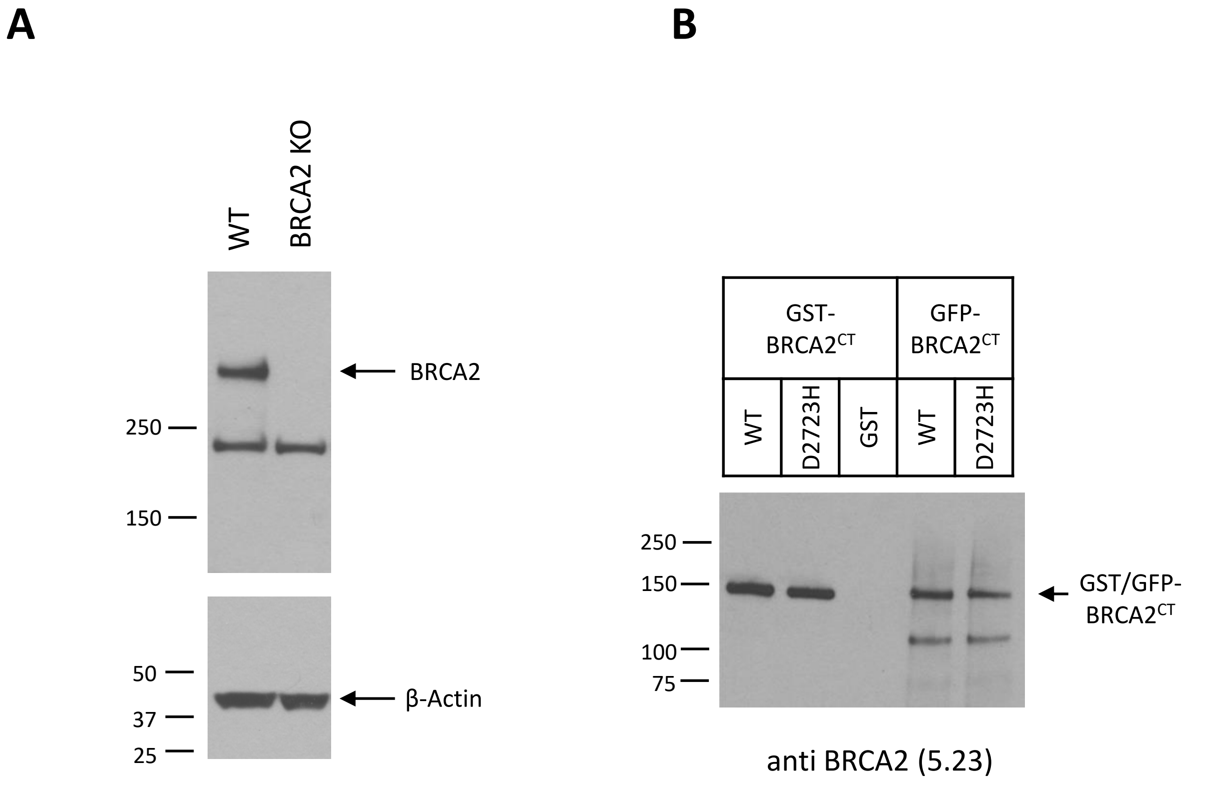

Supplementary Figure 2. (related to Figure 3) (**A**) A western blot showing knock-out of *BRCA2* gene in the BRCA2 KO HeLa cells. (**B**) Purified BRCA2^{CT} fragments detected by western blotting with a monoclonal antibody against the C-terminus of BRCA2 (clone 5.23).

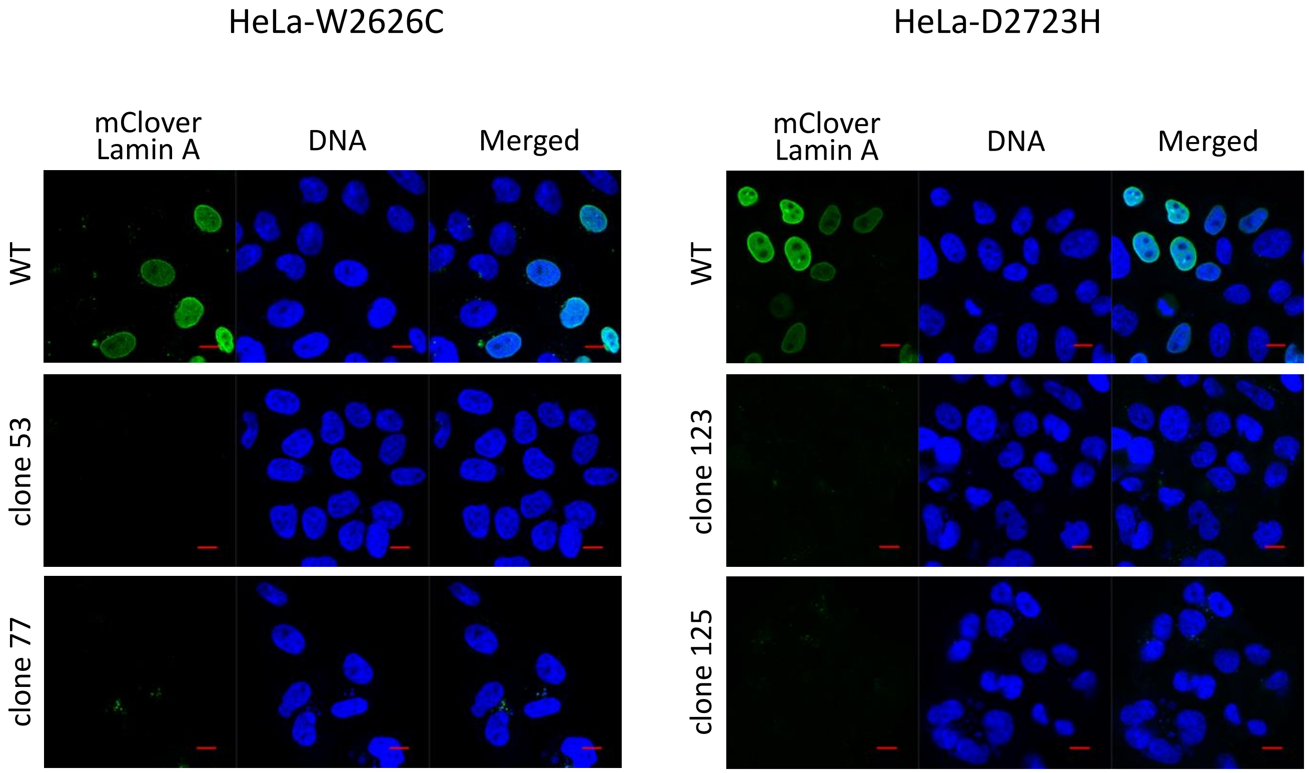

Supplementary Figure 3. (related to Figure 4B). HeLa cells bearing pathogenic BRCA2 DBD mutants are defective in DNA repair by HDR. mClover Lamin A HDR assay. Representative microscopic images of HeLa-W2626C and HeLa-D2723H cells, transfected with Lamin A targeting sgRNA and mClover Lamin A donor constructs and imaged 72 hours later. Scale bar, 10µm.

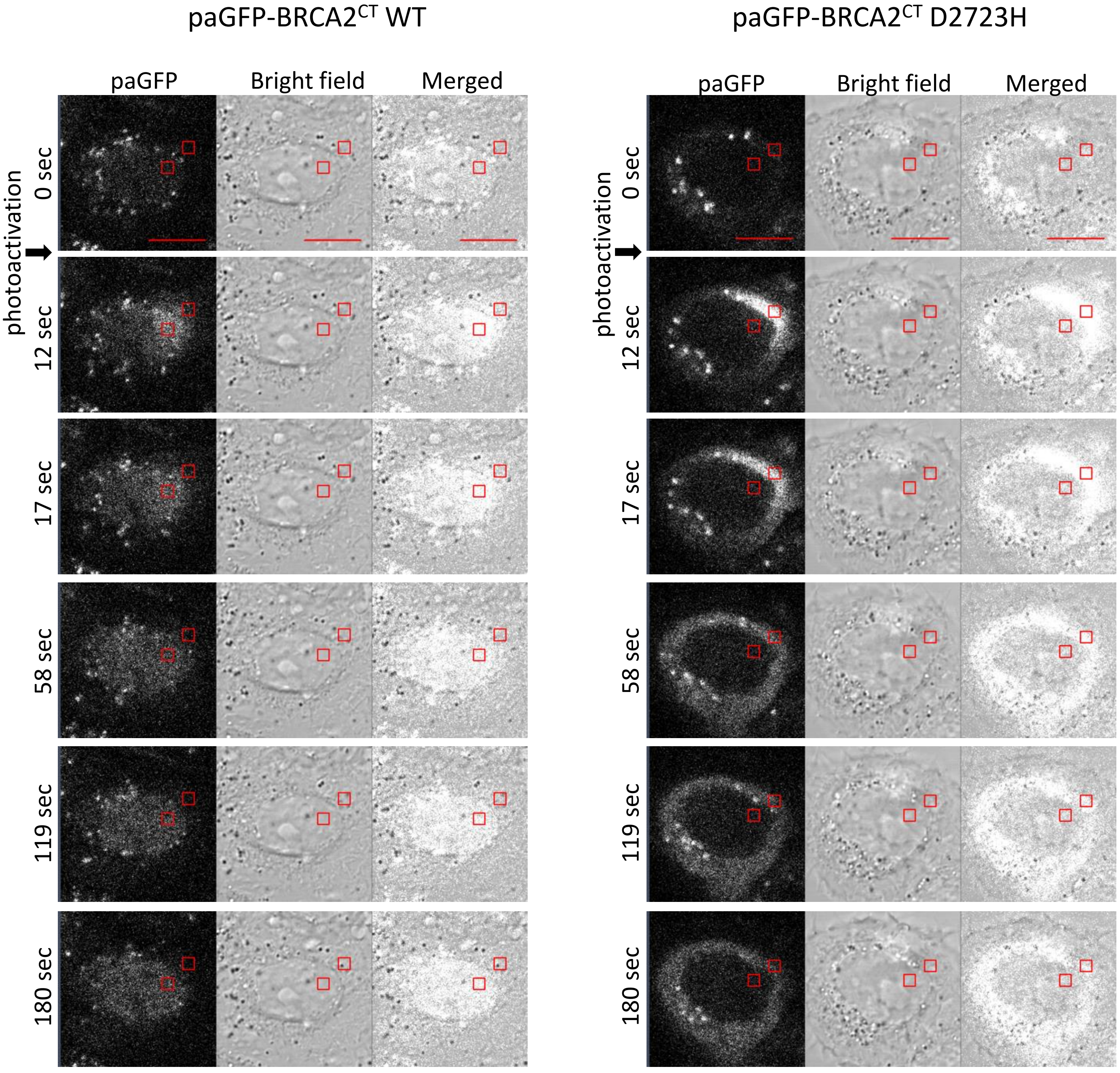

Supplementary Figure 4. (related to Figure 5A) Nuclear import defect of pathogenic BRCA2 DBD mutants. Nuclear import assay using paGFP-BRCA2^{CT} WT and D2723H. HeLa cells expressing paGFP-BRCA2^{CT} fragments were followed by live imaging after cytosolic photoactivation. Red squares mark the area in the cytosol and the nucleus for photoactivation and fluorescence measurement. Representative images from selected frames are shown. Scale bar, 10µm.

Supplementary Table. 1 (related to Figure 1A) Clinical significance of BRCA2 mutations listed in ClinVar. 9,085 variants classified by molecular consequence (mutation type) and clinical significance, out of total 12,684 BRCA2 variants listed in ClinVar, are shown.

	**Mutation type (Molecular consequence)**				
**Clinical significance**	**Frame shift**	**Nonsense**	**Splice site**	**Missense**	**Sum**
**Conflicting interpretations**	6	5	8	531	550
**Benign**	1	1	0	148	150
**Likely benign**	12	4	0	94	110
**Uncertain significance**	11	3	9	4607	4630
**Likely pathogenic**	82	35	96	25	238
**Pathogenic**	2294	911	165	37	3407
**Sum**	2406	959	278	5442	9085

truncated proteins

(Data Downloaded on 2020.07.27)

Supplementary Table. 2 (related to Figure 1B) Clinical significance of BRCA2 missense mutations.

143 benign and pathogenic variants reviewed by the expert panel are shown.

Protein change	Clinical significance (Last reviewed)
R18H	Benign(Last reviewed: Aug 10, 2015)
Y42C	Benign(Last reviewed: Aug 10, 2015)
N56T	Benign(Last reviewed: Aug 10, 2015)
A75P	Benign(Last reviewed: Aug 10, 2015)
P168T	Benign(Last reviewed: Aug 10, 2015)
S326R	Benign(Last reviewed: Aug 10, 2015)
P375S	Benign(Last reviewed: Aug 10, 2015)
S384F	Benign(Last reviewed: Aug 10, 2015)
L452I	Benign(Last reviewed: Aug 10, 2015)
E462G	Benign(Last reviewed: Aug 10, 2015)
K513R	Benign(Last reviewed: Aug 10, 2015)
C554W	Benign(Last reviewed: Aug 10, 2015)
T582P	Benign(Last reviewed: Aug 10, 2015)
G602R	Benign(Last reviewed: Aug 10, 2015)
T630I	Benign(Last reviewed: Aug 10, 2015)
P655R	Benign(Last reviewed: Aug 10, 2015)
D806H	Benign(Last reviewed: Aug 10, 2015)
V894I	Benign(Last reviewed: Aug 10, 2015)
L929S	Benign(Last reviewed: Aug 10, 2015)
N987I	Benign(Last reviewed: Aug 10, 2015)
L1019V	Benign(Last reviewed: Aug 10, 2015)
N1102Y	Benign(Last reviewed: Aug 10, 2015)
S1172L	Benign(Last reviewed: Aug 10, 2015)
R1190W	Benign(Last reviewed: Aug 10, 2015)
G1194D	Benign(Last reviewed: Aug 10, 2015)
N1228D	Benign(Last reviewed: Aug 10, 2015)
C1265S	Benign(Last reviewed: Aug 10, 2015)
D1280V	Benign(Last reviewed: Aug 10, 2015)
V1360I, V1306I, V1360L	Benign(Last reviewed: Aug 10, 2015)
I1349T	Benign(Last reviewed: Aug 10, 2015)
T1354M	Benign(Last reviewed: Aug 10, 2015)
C1365Y	Benign(Last reviewed: Aug 10, 2015)
Q1396R	Benign(Last reviewed: Aug 10, 2015)
D1420Y	Benign(Last reviewed: Aug 10, 2015)
F1524V	Benign(Last reviewed: Aug 10, 2015)
G1529R	Benign(Last reviewed: Aug 10, 2015)
E1537K	Benign(Last reviewed: Aug 10, 2015)
K1690N	Benign(Last reviewed: Aug 10, 2015)
S1733F	Benign(Last reviewed: Aug 10, 2015)
G1771D	Benign(Last reviewed: Aug 10, 2015)
P1819S	Benign(Last reviewed: Aug 10, 2015)
L1904V	Benign(Last reviewed: Aug 10, 2015)
H1918Y	Benign(Last reviewed: Aug 10, 2015)
I1929V	Benign(Last reviewed: Aug 10, 2015)
R2034C	Benign(Last reviewed: Aug 10, 2015)
N2048I	Benign(Last reviewed: Aug 10, 2015)
H2074N	Benign(Last reviewed: Aug 10, 2015)
R2108H	Benign(Last reviewed: Aug 10, 2015)
N2113S	Benign(Last reviewed: Aug 10, 2015)
T2250A	Benign(Last reviewed: Aug 10, 2015)
I2285V	Benign(Last reviewed: Aug 10, 2015)
Q2384K	Benign(Last reviewed: Aug 10, 2015)
N2436I	Benign(Last reviewed: Aug 10, 2015)
K2472T	Benign(Last reviewed: Aug 10, 2015)
T2515I	Benign(Last reviewed: Aug 10, 2015)
A2717S	Benign(Last reviewed: Aug 10, 2015)
K2729N	Benign(Last reviewed: Aug 10, 2015)
R2842H	Benign(Last reviewed: Aug 10, 2015)
R2888C	Benign(Last reviewed: Aug 10, 2015)
V2969M	Benign(Last reviewed: Aug 10, 2015)
R2973C	Benign(Last reviewed: Aug 10, 2015)
V3079I	Benign(Last reviewed: Aug 10, 2015)
Y3098H	Benign(Last reviewed: Aug 10, 2015)
D3170G	Benign(Last reviewed: Aug 10, 2015)
C3198R	Benign(Last reviewed: Aug 10, 2015)
T3349A	Benign(Last reviewed: Aug 10, 2015)
N289H	Benign(Last reviewed: Jan 12, 2015)
N372H	Benign(Last reviewed: Jan 12, 2015)
M784V	Benign(Last reviewed: Jan 12, 2015)
N991D	Benign(Last reviewed: Jan 12, 2015)
C1290Y	Benign(Last reviewed: Jan 12, 2015)

Protein change	Clinical significance (Last reviewed)
I1364L	Benign(Last reviewed: Jan 12, 2015)
T1414M	Benign(Last reviewed: Jan 12, 2015)
D1902N	Benign(Last reviewed: Jan 12, 2015)
T1915M	Benign(Last reviewed: Jan 12, 2015)
K2339N	Benign(Last reviewed: Jan 12, 2015)
H2440R	Benign(Last reviewed: Jan 12, 2015)
A2466V	Benign(Last reviewed: Jan 12, 2015)
I2944F	Benign(Last reviewed: Jan 12, 2015)
V3244I	Benign(Last reviewed: Jan 12, 2015)
I3412V	Benign(Last reviewed: Jan 12, 2015)
N108H	Benign(Last reviewed: Jun 18, 2019)
Q147R	Benign(Last reviewed: Jun 18, 2019)
Q347R	Benign(Last reviewed: Jun 18, 2019)
P389Q	Benign(Last reviewed: Jun 18, 2019)
E394A	Benign(Last reviewed: Jun 18, 2019)
I505T	Benign(Last reviewed: Jun 18, 2019)
D596H	Benign(Last reviewed: Jun 18, 2019)
T598A	Benign(Last reviewed: Jun 18, 2019)
S599F	Benign(Last reviewed: Jun 18, 2019)
Y600H	Benign(Last reviewed: Jun 18, 2019)
K604E	Benign(Last reviewed: Jun 18, 2019)
A622V	Benign(Last reviewed: Jun 18, 2019)
N900D	Benign(Last reviewed: Jun 18, 2019)
E919K	Benign(Last reviewed: Jun 18, 2019)
D935H	Benign(Last reviewed: Jun 18, 2019)
L996R	Benign(Last reviewed: Jun 18, 2019)
F1192C	Benign(Last reviewed: Jun 18, 2019)
S1424C	Benign(Last reviewed: Jun 18, 2019)
E1593D	Benign(Last reviewed: Jun 18, 2019)
I1851S	Benign(Last reviewed: Jun 18, 2019)
N1878K	Benign(Last reviewed: Jun 18, 2019)
N1880K	Benign(Last reviewed: Jun 18, 2019)
H1918R	Benign(Last reviewed: Jun 18, 2019)
D1923A	Benign(Last reviewed: Jun 18, 2019)
T2097M	Benign(Last reviewed: Jun 18, 2019)
R2108C	Benign(Last reviewed: Jun 18, 2019)
S2152Y	Benign(Last reviewed: Jun 18, 2019)
D2312V	Benign(Last reviewed: Jun 18, 2019)
R2318Q	Benign(Last reviewed: Jun 18, 2019)
G2353R	Benign(Last reviewed: Jun 18, 2019)
L2396F	Benign(Last reviewed: Jun 18, 2019)
K2411T	Benign(Last reviewed: Jun 18, 2019)
R2502H	Benign(Last reviewed: Jun 18, 2019)
L2512F	Benign(Last reviewed: Jun 18, 2019)
D2665G	Benign(Last reviewed: Jun 18, 2019)
V2728I	Benign(Last reviewed: Jun 18, 2019)
A2770T	Benign(Last reviewed: Jun 18, 2019)
S2835P	Benign(Last reviewed: Jun 18, 2019)
A2912T	Benign(Last reviewed: Jun 18, 2019)
S2922G	Benign(Last reviewed: Jun 18, 2019)
T3013I	Benign(Last reviewed: Jun 18, 2019)
K3015E	Benign(Last reviewed: Jun 18, 2019)
K3059E	Benign(Last reviewed: Jun 18, 2019)
P3292L	Benign(Last reviewed: Jun 18, 2019)
T3374I	Benign(Last reviewed: Jun 18, 2019)
H2116R	Benign(Last reviewed: Sep 28, 2016)
I2490T	Benign(Last reviewed: Sep 28, 2016)
A2951T	Benign(Last reviewed: Sep 28, 2016)
W2626C	Pathogenic(Last reviewed: Aug 10, 2015)
I2627F	Pathogenic(Last reviewed: Aug 10, 2015)
R2659T	Pathogenic(Last reviewed: Aug 10, 2015)
E2663V	Pathogenic(Last reviewed: Aug 10, 2015)
T2722R	Pathogenic(Last reviewed: Aug 10, 2015)
D2723H	Pathogenic(Last reviewed: Aug 10, 2015)
D2723G	Pathogenic(Last reviewed: Aug 10, 2015)
G2748D	Pathogenic(Last reviewed: Aug 10, 2015)
R3052W	Pathogenic(Last reviewed: Aug 10, 2015)
M1I, M231I	Pathogenic(Last reviewed: Jun 18, 2019)
R2336H	Pathogenic(Last reviewed: Jun 18, 2019)
R2659G	Pathogenic(Last reviewed: Jun 18, 2019)
I2675V	Pathogenic(Last reviewed: Jun 18, 2019)
N3124I	Pathogenic(Last reviewed: Jun 18, 2019)

Supplementary Table. 3 (related to MATERIALS AND METHODS) sgRNA and HDR template sequences for genome editing. sgRNA and HDR template sequences used in this study to generate HeLa-W2626C and HeLa-D2723H cells. Additional silent mutations to create ClaI and SacI sites were introduced in the HDR templates for screening of clones by restriction enzyme digestion of genomic PCR products.

	sgRNA	HDR template
W2626C	GGGTTTATAATCA CTATAGA	TTGTTCAGGGCTCTGTGTGACACTCCAGGTGTGGATCC AAAGCTTATTTCTAGAATTTGGGTTTATAATCACTATCG ATGCATCATATGGAAACTGGCAGCTATGGAATGTGCCT TTCCTAAGGAATTTGCTAATAGATGCCTAAGCCCAGAA AGGGTGCTTCTTCAACTAAAAT (ClaI)
D2723H	GCCATTATTGAAC TTACAGA	AAGAATAATCTTCTGACCAACTGTCAGTCTGCCATTCTT TAAGACAGCTAAGAGGGGAGGATCTAACTGGGCCTTA ACAGCATACCACCCATGTGTGAGCTCAATAATGGCCAC TTTTTGGGTATCTGCACTACTAGTTTTATTGCTAGAAGT TTCAGATATATTTGCGCTCAATGAAAT (SacI)
